# Making the Grade in a Portfolio-Based System: Student Performance and the Student Perspective

**DOI:** 10.3389/fpsyg.2013.00155

**Published:** 2013-04-02

**Authors:** Amy S. Nowacki

**Affiliations:** ^1^Department of Quantitative Health Sciences, Lerner College of MedicineCleveland Clinic, Cleveland, OH, USA

**Keywords:** assessment, portfolio, grading, student performance, student perspective

## Abstract

Assessment is such an integral part of the educational system that we rarely reflect on its value and impact. Portfolios have gained in popularity, but much attention has emphasized the end-user and portfolio assessment. Here we focus on the portfolio creator (the student) and examine whether their educational needs are met with such an assessment method. This study aims to investigate how assessment practices influence classroom performance and the learning experience of the student in a graduate education setting. Studied were 33 medical students at the Cleveland Clinic Lerner College of Medicine of Case Western Reserve University, a program utilizing a portfolio-based system. The students may elect to simultaneously enroll in a Masters program; however, these programs employ traditional letter grades. Thus creating a unique opportunity to assess 25 portfolio only (P) students and 8 portfolio and grade (PG) students concurrently taking a course that counts for both programs. Classroom performance was measured *via* a comprehensive evaluation where the PG students scored modestly better (median total scores, 72% P vs. 76% PG). Additionally, a survey was conducted to gain insight into student’s perspective on how assessment method impacts the learning experience. The students in the PG group (those receiving a grade) reported increased stress but greater affirmation and self-assurance regarding their knowledge and skill mastery. Incorporation of such affirmation remains a challenge for portfolio-based systems and an area for investigation and improvement.

## Introduction

Assessment refers to the process of forming a judgment about the quality and extent of student achievement or performance, and therefore inferring a judgment about the learning that has taken place (Sadler, 2005). Assessment is such an integral part of the educational system that we often take it for granted. Yet there is tremendous variability in assessment practices, and indeed even the assumed purpose of assessment varies a great deal. For many years, the primary objective of higher education has been to make students knowledgeable within a particular domain. Building a basic knowledge store was the central issue. Recent developments within society, such as the increasing production of new scientific knowledge and the use of modern communication technology, have encouraged us to implement new methods that are in line with these developments (Dochy and McDowell, 1997). These new methods, such as case-based and problem-based learning, are directed toward producing highly knowledgeable individuals, but do also stress problem-solving skills, professional skills, and authentic learning, i.e., learning in real-life contexts (Dochy et al., 1999). To adapt, many institutions have adopted competency-based assessment systems such as portfolios.

Numerous benefits have been described regarding portfolio-based assessment systems. In particular for medical education, the assessment of learning outcomes not easily assessed by other methods such as personal growth, self-directed learning, reflective ability, and professionalism (Friedman Ben David et al., 2001). What are the major issues in portfolio-based learning and assessment? Despite the many advantages of portfolio-based learning, there are particular issues that may prove problematic. Some concerns include the difficulty in assessing portfolios (Snadden and Thomas, 1998) due to their highly personal nature and limited points of objectivity that allow comparisons to be made between students. Assessment is also labor intensive and requires careful reading and response to a learner’s objectives and evidence of whether they have been met (Challis, 1999). These concerns, however, emphasize the struggle in using portfolios primarily focusing on the end-user, but what about the needs of the portfolio creator? Are students receiving all that they need and desire out of the portfolio?

This article investigates the impact of assessment method, portfolio only (P) vs. portfolio and grade (PG), on student performance and student perception of their learning experience. Does the addition of a letter grade impact student performance? Are the needs of students being met within a portfolio system alone or is there value-added/subtracted with grade supplementation?

## Materials and Methods

The Cleveland Clinic Lerner College of Medicine (CCLCM) implements a portfolio system for assessment where students receive feedback from faculty and peers which can be used as evidence of skill mastery or deficiency (Dannefer and Henson, 2007). Thus there are no letter grades or class ranks to document student performance; rather each student receives a number of qualitative assessments of their performance. Based on the premise that assessment should be to enhance learning, feedback is provided in relation to nine broad-based competencies (e.g., medical knowledge, research, clinical skills, clinical reasoning, communication, professionalism, health care systems, personal development, and reflective practice) essential for physician investigators. Students generate and submit periodically their portfolios to a medical student promotions and review committee composed of clinical and basic science faculty members who determine students’ eligibility for promotion to the next phase of the curriculum and graduation from medical school.

All CCLCM students must enroll in the Introductory Biostatistics course as part of the required research curriculum. This 9 week course is taught in a problem-based learning style described in detail elsewhere (Nowacki, 2011). During the most recent course offering, 33 CCLCM students were enrolled in the Introductory Biostatistics course. A subset of students has elected to also enroll in one of several Masters Degree programs offered through Case Western Reserve University. These programs all require the completion of an Introductory to Biostatistics course and thus the course can be double counted for both degree requirements. The only stipulation is that students must receive a letter grade upon completion for the Masters Programs. Thus, these students are required to complete four additional assignments upon which their letter grade is based. Each assignment consists of five or six contextual problems based on the medical literature where students are asked to explain the authors’ selection of analytic techniques, perform analysis of published data, identify errors in reported results, or recognize techniques that would have improved the analysis. Other students in the class not requiring a grade also have access to these assignments, but are not obliged to complete them. During the most recent course offering, eight CCLCM students were enrolled in a Masters Degree program.

### Student performance

On the last day of class, comprehensive skills assessment was completed by all attending (32 out of 33 students). This assessment was not announced as the goal was to evaluate the concept mastery obtained by the self-directed learners and not evaluate how well the students could prepare for a final evaluation. The students were given 1 h to complete a nine question assessment which incorporated both calculation and conceptual based questions. The assessment had a possible 100 points and each student was given a score for the purposes of this investigation. Calculation questions comprised four of the nine and scores could range from 0 to 44. These questions required students to compute probabilities using both discrete and continuous distributions, compute standard errors, and implement Bayes theorem. Conceptual questions comprised five of the nine and scores could range from 0 to 56. These questions investigated definitional understanding with true/false, identification of appropriate statistical tests for research scenarios, and explanations of constructs such as sampling variation, hypothesis testing, and components of sample size determination. All students were provided with the solutions the next day providing an opportunity for reflection and self-assessment of performance. Dot plots along with mean bars summarize the total score, calculation and conceptual subscale scores for the P and PG groups.

### Student perception

A brief survey was designed to gather information regarding student’s perception of how the assessment method they received (P vs. PG) impacted various aspects of the course. All CCLCM students enrolled in the most recent offering of the Introductory Biostatistics course received an email invitation to complete the survey. The survey introduction explained that the goals are to: (1) evaluate and improve the assessment of the second year clinical research block courses; and (2) gain insight into the student perspective on how various assessment methods impact learning and the classroom experience. The survey invitation was sent to 33 students with 26 (79%) participating. Students were asked to score their opinion from 0 to 100 (Table 1) regarding 19 constructs of learning and the classroom experience. Constructs were selected with anticipation that some would show benefit, some detriment, and others remain unchanged with the addition of a letter grade (Jason and Westberg, 1979). Results of the survey were captured in a REDCap secure database. Scores are summarized and presented as mean (minimum, maximum) and Wilcoxon rank-sum tests compare scores among the P and PG groups. Focus is placed on those constructs achieving at least marginal significance (*p* ≤ 0.10) and hence the largest relative effect as this is a small sample and the intention of the analysis is hypothesis generation. This survey was conducted with Cleveland Clinic IRB approval.

**Table 1:** **Student perceptions of the impact of assessment method on learning and the classroom experience**.

Construct	Scale 0 to 100		Portfolio Only	Portfolio and Grade	*p*-value
Personal motivation for learning	Negatively impact	Positively impact	72 (50, 100)	83 (75, 100)	0.19
Preparation for class sessions	Decreased	Increased	66 (40, 100)	84 (70, 100)	0.03
Willingness to seek help from instructor or TA	Decreased	Increased	68 (49, 100)	83 (50, 100)	0.07
Personal Sense of achievement	Negatively impact	Positively impact	62 (30, 90)	81 (60, 100)	0.04
Enjoyment of the course	Negatively impact	Positively impact	80 (25, 100)	65 (35, 100)	0.11
Demonstration of abilities to others	Negatively impact	Positively impact	64 (40, 88)	77 (60, 90)	0.06
Course-associated stress	Decreased	Increased	30 (0, 81)	69 (50, 100)	0.002
View of student assessment	Subjective	Standardized	51 (20, 85)	69 (40, 100)	0.14
Ability to focus on aspects of summer block that I felt important	Negatively impact	Positively impact	82 (60, 100)	71 (50, 100)	0.21
Proving skill mastery to yourself	Negatively impact	Positively impact	59 (30, 85)	79 (59, 100)	0.01
View of instructor	Negatively impact	Positively impact	74 (50, 100)	73 (50, 100)	0.89
Feedback viewed as being	critical	Constructive	73 (48, 100)	78 (60, 100)	0.52
Improvement in myself	Negatively impact	Positively impact	75 (59, 100)	75 (50, 100)	0.93
The learning environment	Competitive	Collaborative	89 (55, 100)	81 (56, 100)	0.28
Ability to understand and accept my strengths and weaknesses	Negatively impact	Positively impact	75 (39, 100)	70 (50, 100)	0.52
Learning to provide feedback to others	Negatively impact	Positively impact	77 (52, 100)	60 (50, 75)	0.01
I view learning as a:	Mandate	Opportunity	84 (52, 100)	84 (70, 100)	0.86
Sufficiently learned what I needed to:	Unsure	Confident	68 (24, 94)	85 (72, 95)	0.02
Overall, with respect to my learning, the assessment approach seemed to:	Impede	Foster	73 (33, 100)	78 (50, 100)	0.56

^*^Reported as mean (min, max); *p*-value from Wilcoxon rank-sum test.

## Results

**Figure 1:**
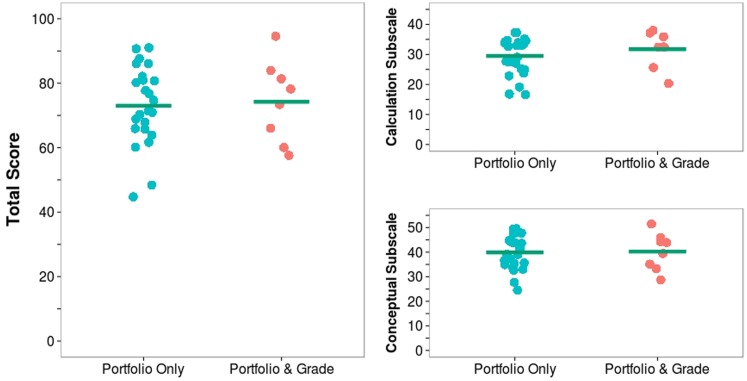
**Student performance on comprehensive course assessment by assessment method**. PG students score modestly higher on the total score. PG students score higher on the calculation-based subscore. P and PG students score similarly on the conceptual-based subscore. Horizontal green line represents group mean. Points are jittered for legibility.

Of the 32 students completing the comprehensive skills assessment, 24 were P students, and 8 were PG students. Figure 1 summarizes the student performance results. There was a modest difference among the two groups on their median total scores (72% P vs. 76% PG) with students assessed by both portfolio and grade scoring higher. Both groups scored similarly on the conceptual-type questions with the observed difference coming as a result of the PG students scoring higher on the calculation-type questions. The PG students also completed the assessment in less time than the P students (median rank 11 vs. 19).

Of the 26 students completing the student perceptions survey, 19 were P students, and 7 were PG students. Table 1 presents the student perception results by assessment methodology. Interestingly, with the addition of a grade to the portfolio assessment methodology students reported numerous positive constructs such as: increased preparation for class sessions, increased willingness to seek help, a positively impacted sense of personal achievement, a positively impacted capability to demonstrate their abilities to others, a positively impacted capacity to prove skill mastery to self, and more confidence that they sufficiently learned what they needed to. Additionally, students also reported that the addition of a grade to the portfolio assessment methodology increased course-associated stress and negatively impacted the ability to learn to provide feedback to others.

## Discussion

All results must be viewed as hypothesis generating as the small sample sizes limit the robustness of the results. With this in mind, we have limited the statistical inference and focused on descriptive analysis of this cohort. Despite the limitations, some interesting findings have emerged and are discussed below.

The fact that the students receiving a grade in addition to the portfolio assessment scored modestly higher on the course final evaluation is not surprising in that more is at stake for these individuals. They will have a transcript that displays only a limited number of course grades associated with their Masters Degree Program and none from their medical degree program. Thus, the impact of each grade is magnified as this transcript is an important piece of evidence requested during the residency interview process. If anything, it is surprising that the difference in performance among the groups was not more substantial. One can speculate that the higher performance on calculation-type questions is due to the fact that the PG students had to actually carry out more calculations and practice more by completing the four additional assignments without sole reliance on statistical software. Others too have found that working a small example by hand/calculator and then using a computer package helps teach the concepts and build confidence in the computer approach (Steinhorst and Keeler, 1995). Not being able to parse out the role of the additional assignments is a study limitation. As the P students had access to these assignments, informally many reported simply reviewing them without actually attempting the questions. Unfortunately no data was available regarding actual use. The equivalent performance on the conceptual-type questions can be viewed as a success of the course format designed for the portfolio focused student. To achieve such a competitive level of conceptual mastery without the high stakes of a letter grade implies that students are finding other modes of motivation. Perhaps it is the special cohort of medical students who attend a research focused school, or the careful alignment of the course objectives with research activities the student’s will be performing both in the remainder of the curriculum and on the job, or maybe the influence of their respected mentors.

The result that students who additionally received a grade felt that it positively impacted their sense of personal achievement, capability to demonstrate their abilities to others, capacity to prove skill mastery to self, and increased their confidence that they sufficiently learned what they needed to aligns with the idea that objective measures of performance provide a “safety blanket” for students self-assurance. Here the grade assigned for the course actually reaffirmed student perspective on the portfolio process. This was similarly observed (Altahawi et al., 2012) where students cited their score on the USMLE step 1 exam (first national exam in a series required for medical licensure) as evidence of the success of their non-test-based portfolio system. Thus the students turn to an objective test score (a successful step 1 score) to provide affirmation regarding their knowledge and alluding to an uncertainty in the portfolio process alone. It appears that this cohort of students also felt that the objective scores on the four assignments and the end-of-course grade provide confirmation of skill mastery at a level not perceived by the P students. Similarly aligned is the common method of “grading” portfolios so as to assist in the decision making process of student promotion. Ironically there is a desire to assign a standardized measure to confirm sufficient student performance, an example described in (Friedman Ben David et al., 2001), when the portfolio itself is often implemented as a means to circumventing such a process.

The stress experienced by medical students related to grades and performance has been documented (Stewart et al., 1995). This PG student cohort also report increased stress levels not reported by the P students. This is probably tied to both the issuing of a letter grade in this course and the structure of this graduate program where students receive only a handful of grades (typically 3–5) for their transcript and thus each carries significant weight. Remarkably, despite the increased stress, the PG students report similar feelings that the assessment approach fosters their learning (means: 78 PG vs. 73 P). Thus the stakes are higher but apparently the payoff is considered worthwhile.

It appears from the student responses that they are satisfied within the portfolio system and that it provides them with necessary feedback for reflection and personal educational growth. The addition of a grade to the portfolio system, while increasing stress levels, provides these students with a self-reported desirable affirmation of their skills and knowledge. Incorporation of such affirmation remains a challenge for portfolio based systems and an area for investigation and improvement.

## Conflict of Interest Statement

The authors declare that the research was conducted in the absence of any commercial or financial relationships that could be construed as a potential conflict of interest.
